# Narrative Research in an Aging Society: A Multidisciplinary Analysis for Older People in Taiwanese Film Yi Yi

**DOI:** 10.1093/geroni/igaf122.3352

**Published:** 2025-12-31

**Authors:** Sheng-Chin Hsu

**Affiliations:** Ming Chuan University, Taipei, Taipei, Taiwan (Republic of China)

## Abstract

This study adopts narrative methods to investigate the impact of the grandmother character, who requires long-term care due to an accident in the Taiwanese film Yi Yi, on the overall narrative, other characters, and the development of the narrative. This research analyzes the narrative composition of the film’s text and then validates and interprets it theoretically from the perspectives of narrative research and gerontology. Additionally, this paper incorporates relevant discussions of Yang De-chang’s creative context, attempting to understand through an interdisciplinary approach how the long-term care older people in Taiwanese films impacts other family members and how family risks are formed in the narrative development. The purpose of this study is to illustrate that the older people who needs long-term care due to an accident, although silent, influences the film’s narrative structure and the new decisions among family members. Through interdisciplinary knowledge analysis of narrative theory and gerontology, this study finds that the family in the story is the current majority nuclear family in Taiwan. In a state of fewer people, the family support network is weakened, highlighting the challenges of long-term care for nuclear families in modern society. The knowledge spectrum of gerontology expands the path of narrative analysis for understanding the aging society, which reminds contemporary researchers that understanding the aging society must develop interdisciplinary methods and comprehensive explanations to provide culturally diverse solutions to the problems of the aging society and achieve harmony between generations.

